# A surgeon, a doctor and a baby – combining parenthood with a medical career

**DOI:** 10.1515/iss-2018-0027

**Published:** 2019-04-30

**Authors:** Steven C. Herath, Esther Herath

**Affiliations:** Department of Trauma, Hand and Reconstructive Surgery, Saarland University, D-66421 Homburg/Saar, Germany; Department of Trauma, Hand and Reconstructive Surgery, Saarland University, Homburg/Saar, Germany; Department of Medicine II, Saarland University, Homburg/Saar, Germany

**Keywords:** childcare, health professionals, parenthood, working conditions, young surgeons

## Abstract

Double-physician couples being parents have been shown to face greater difficulties in combining their private and professional lives when compared to other couples. In the present study, we aimed to analyze how double-physician couples manage to arrange their roles in their private and professional lives and how compatible their individual idea of being a mother or a father is with their career as a physician. Fifteen couples being parents and consisting of either two surgeons or a surgeon and a nonsurgeon were asked to participate in a survey to determine the average maternity or paternity leave, the reduction of hours worked per week after the birth of a child, and the need for professional childcare and additional support in childcare from relatives or babysitters per week. Furthermore, the couples were asked to mark on a six-item Likert scale how compatible their professional life is with their idea of being parents. The average maternity or paternity leave was 13 ± 2 months per child and the mean reduction of hours worked per week was 30 ± 12%. The couples made use of professional childcare for 41 ± 6 h/week on average and needed additional support in childcare from relatives or babysitters for 5 ± 3 h/week. On the Likert scale from “completely incompatible (0)” to “perfectly compatible (5)”, the mean compatibility of professional and private lives was rated 2.5 ± 1.1. Becoming parents significantly influences the professional and private lives of double-physician couples. The relatively low compatibility of double-physician couples’ private and professional lives might lead to relevant work-home conflicts. Such conflicts have been proven to be associated with surgeons not recommending surgery as a career. Therefore, efforts should be made to improve the compatibility of parenthood and a medical career.

## Introduction

If you are a young surgeon and you fall in love with a young doctor or another young surgeon, you will soon get to know the difficulties of synchronizing duty rosters and leave days. If things get more serious, you will learn that scheduling a wedding seems impossible for roughly the next 12s months and that about 30% of your friends won’t be able to attend the party because they are also health-care professionals and cannot get that particular day off. Although in the authors’ opinion the mentioned difficulties are more or less annoying, the scenario of two physicians becoming parents has the potential to shake the foundations of both partners’ professional and private lives.

About 30% of male surgeons and up to 50% of female surgeons have a domestic partner who is also a physician [1], [2], [3], [4]. It has been demonstrated that surgeons whose spouse is also a physician have to face greater difficulties in combining their private and professional lives when compared to couples consisting of a physician and a nonphysician [5]. The international literature states that the risk for surgeons’ “work-home conflicts” is independently associated with the number of hours worked per week and having children [6]. Currently, with the “Generation Y” doing the ward rounds and getting scrubbed at the theatre, the term “work-life balance” seems to gain importance among younger surgeons and the parental satisfaction of physicians has been subject to scientific research [7], [8].

Experiencing the strong influence of parenthood on the life of a young double-physician couple themselves, the authors aimed to analyze how other physician couples with children manage to arrange their roles in private and professional lives. Furthermore, we aimed to evaluate how satisfied the couples are with their individual solutions.

## Methods

To assess whether and how intensively the roles “parents” and “physicians” interact with each other, we asked 15 couples being parents and consisting of either two surgeons or a surgeon and another physician to participate in a survey. The participants were working in 10 different institutions. Exclusively younger health-care professionals who have been working for 10 years or less were included in the study.

We aimed to analyze how long the average maternity or paternity leave had been and whether working hours have been reduced after becoming parents. Furthermore, we collected data on how many hours per week the physician families need support in childcare from either a kindergarten or other persons, such as babysitters, relatives, or day nannies. In addition, the participants were asked to mark on a six-item Likert scale from “completely incompatible (0)” to “perfectly compatible (5)” how compatible their professional life is with their individual idea of being a mother or a father.

In addition to the overall analysis, we compared two-surgeon couples to couples consisting of a surgeon and a nonsurgeon concerning the aforementioned variables.

For statistical analyses, Student’s t-test was used after testing the data for normal distribution (Kolmogorov-Smirnov test) and equal variance (*F* test). p<0.05 was considered to indicate a significant difference. All statistical analyses were performed using the SigmaPlot^®^ software package (Systat Software, Inc., San Jose, CA, USA).

## Results

All couples who were asked to participate in the survey returned a complete questionnaire. The participants were 15 men and 15 women, being parents of a total of 26 children. Twenty one (70%) participants were surgeons and 9 (30%) were physicians of other specialties. Six couples consisted of two surgeons and nine couples consisted of a surgeon and a nonsurgeon. The mean duration of maternity or paternity leave was 13±2 months per child (range, 9–18 months). In all couples except for one, the weekly working hours were reduced after the birth of a child. The couple who did not reduce the hours worked per week consisted of a surgeon and a nonsurgeon. The average reduction of hours worked per week was 30±12% (range, 20–50%), and for all couples, it was only one partner who reduced their working hours. The mean need for professional childcare was 41±6 h/week (range, 25–50 h). Ten families (67%) made use of additional support in childcare from family members or babysitters (5±3 h/week; range, 1.5–10 h). On the six-item Likert scale designed to rate the compatibility of each physician’s professional life with their individual idea of being a mother or a father, the average score was 2.5±1.1 (Table 1).

**Table 1:** Influence of parenthood on the professional and private lives of couples consisting of either two surgeons or a surgeon and a nonsurgeon.

Variable	All couples (n=15)	Two-surgeon couples (n=6)	Mixed couples (n=9)	p (two-surgeon vs. mixed)
Children (n)	1.7±0.6	1.8±0.4	1.7±0.7	0.76
Leave per child (months)	13±2	13±3	13±2	1.00
Reduction of hours worked per week (%)	30±12	25±6	34±14	0.17
Childcare per week (h)	41±6	43±5	40±7	0.38
Additional support per week (h)	5±3	3±1	6±3	0.04
Compatibility of professional and private lives (Likert scale 0–5)	2.5±1.1	2.8±0.4	2.3±1.4	0.42

Data are mean±standard deviation.

When comparing two-surgeon couples to couples consisting of a surgeon and a nonsurgeon, we found that the latter made significantly more use of support in childcare from relatives or babysitters in addition to professional childcare (p=0.04). Concerning all other aforementioned variables, there were no significant differences between two-surgeon couples and mixed physician couples (Table 1).

## Discussion

Just like other people in demanding professions with high levels of responsibility, physicians are confronted with numerous challenges not only in their working life but also in their relationships [9], [10]. Interestingly, relationships of physicians are reported to last longer than those of other professions [11] and double-physician relationships have been shown to bring a significant satisfaction for both partners from the shared professional interest as well as a high engagement in child-rearing [9], [10].

Our data show that becoming parents leads to significant changes not only in the private but also in the professional lives of double-physician couples. The maternity or paternity leave in combination with the reported reduction of hours worked per week (Table 1) brings a relevant delay of surgical and medical training and a reduction of hours worked per week significantly delays specialization if training has already been completed. As the mean reduction of hours worked per week was approximately 30%, it has to be assumed that the resulting mean delay of training or specialization is also about 30%.

Furthermore, we found the physician couples to make use of professional childcare for about as much as 100% working week, whereas the average reduction of hours worked per week was markedly lower. This might indicate that the service hours of childcare institutions do not match the working hours of physicians. For example, some hospitals in Germany have a kindergarten available, which is open 7 days/week and 15.5 h/day [7], although there are also university medical centers with more than 2500 employees and a child-care institution that a child can attend for a maximum of 20 h/month. In our opinion, the discrepancy between the availability of and the need for professional childcare is emphasized by the finding that two thirds of all couples needed additional support in childcare from relatives or other persons such as babysitters and nannies.

Although there were no differences concerning all other variables, couples consisting of a surgeon and a nonsurgeon needed significantly more support from relatives or other persons in addition to professional childcare (Table 1). This can most probably be explained by the different working hours and the different daily routines of surgeons and nonsurgeons.

In our survey, double-physician couples stated the compatibility of their professional and private lives to be relatively low. This is in line with previously published data, stating that surgeons partnered to physicians experience greater difficulties balancing their private and professional lives than surgeons whose spouse is a nonphysician [5]. The resulting work-home conflicts of surgeons partnered to surgeons or physicians have been proven to be associated with symptoms of depression and might lead to surgeons not recommending surgery as a career option to their children [6]. We conclude that all efforts should be made to make the private and professional lives of double-physician couples having children more compatible to preserve surgery and medicine in general as an interesting career option.

Of course, the demands of young surgeons and physicians being parents are widely diversified and very individual. Nevertheless, the authors are convinced that the sum of numerous small and easily implementable changes in daily routines can lead to a significant increase in compatibility of parenthood and a medical career. Big things have small beginnings.

## Limitations

Our study has several limitations. First of all, the sample size is relatively small. However, we feel that the data from 30 participants give a sufficient overview on the influence of parenthood on a medical career. The most important limitation is the lack of a control group consisting of academic couples with a nonmedical profession. This is due to the fact that we designed the study as a proof of concept to analyze the impact of parenthood on the careers of double-physician couples. The comparison to a group of nonphysician couples was beyond the scope of our project and will be addressed in a follow-up study.

## Supplementary Material

The article (iss-2018-0027) offers reviewer assessments as supplementary material.
