# Supplementary material for: A surgeon, a doctor and a baby – combining parenthood with a medical career
Source: Innov Surg Sci. 2019 Apr 30;4(1):31–4. doi: 10.1515/iss-2018-0027 (PMC6754065; doi:10.1515/iss-2018-0027)
Supplement: Supplementary file 1 [file iss-4-20180027-s001.pdf]

Reviewer Assessment

Steven C. Herath* and Esther Herath

A surgeon, a doctor and a baby – combining parenthood with a medical career

https://doi.org/10.1515/iss-2018-0027
Received September 14, 2018; accepted March 21, 2019

***Corresponding author: Steven C. Herath,** Department of Trauma, Hand and Reconstructive Surgery, Saarland University, D-66421 Homburg/Saar, Germany, E-mail: steven.herath@uks.eu

Reviewers' Comments to Original Submission

Reviewer 1: anonymous

Dec 17, 2018

**Reviewer Recommendation Term:**	Accept with Minor Revision
**Overall Reviewer Manuscript Rating:**	85
**Custom Review Questions**	**Response**
Is the subject area appropriate for you?	5 - High/Yes
Does the title clearly reflect the paper's content?	5 - High/Yes
Does the abstract clearly reflect the paper's content?	5 - High/Yes
Do the keywords clearly reflect the paper's content?	5 - High/Yes
Does the introduction present the problem clearly?	5 - High/Yes
Are the results/conclusions justified?	4
How comprehensive and up-to-date is the subject matter presented?	4
How adequate is the data presentation?	5 - High/Yes
Are units and terminology used correctly?	5 - High/Yes
Is the number of cases adequate?	3
Are the experimental methods/clinical studies adequate?	4
Is the length appropriate in relation to the content?	5 - High/Yes
Does the reader get new insights from the article?	4
Please rate the practical significance.	4
Please rate the accuracy of methods.	4
Please rate the statistical evaluation and quality control.	4
Please rate the appropriateness of the figures and tables.	4
Please rate the appropriateness of the references.	4
Please evaluate the writing style and use of language.	4
Please judge the overall scientific quality of the manuscript.	4
Are you willing to review the revision of this manuscript?	Yes

Open Access. © 2019 Herath S.C., Herath E., published by De Gruyter. CC BY This work is licensed under the Creative Commons Attribution 4.0 Public License.

Comments to Authors:

In the present study, the authors present data on a currently burning issue - the combination of parenthood and medical careers. The authors surveyed 15 ‚double-physician' couples about the obstacles they face trying to combine parenthood and a medical career. The survey involved questions about the length of maternity or paternity leave, the reduction of working hours, the amount of time the children had to be cared for by other people or institutions than the parents themselves and the compatibility of their personal to their professional lives.

The present study is well conducted and of good readability. There only remain some minor comments:

1. Title: The title is well chosen and quickly arouses interest, while also informative.

2. The abstract is within the desired word count and summarizes the manuscript in brief.

3. The introduction is concise and states the purpose of the study.

4. The layout of the study and the statistic tests applied are comprehensible and adequate. Selection of participants, study protocol and statistics are described.

5. Interestingly the working hours were reduced in all couples except for one couple. Did this one couple consist of two surgeons or was this an ‚inter-professional' couple? Also, did the mother or the father or both reduce the working hours?

6. In the discussion the authors state, that the use of professional child care exceeded the number of reported working hours. The conclusion, that the service hours of child care institutions do not match the working hours of physicians seems to be a reasonable point. Another factor could be the extra work load and working hours physicians especially in university hospitals face.

7. References are clearly presented and represent the current state of literature. References should be formatted so that indent is equal.

All in all a very interesting and enjoyable manuscript!

Reviewer 2: anonymous

Mar 05, 2019

**Reviewer Recommendation Term:**	Revise with Major Modifications
**Overall Reviewer Manuscript Rating:**	-
**Custom Review Questions**	**Response**
Is the subject area appropriate for you?	4
Does the title clearly reflect the paper's content?	4
Does the abstract clearly reflect the paper's content?	5 - High/Yes
Do the keywords clearly reflect the paper's content?	5 - High/Yes
Does the introduction present the problem clearly?	4
Are the results/conclusions justified?	4
How comprehensive and up-to-date is the subject matter presented?	5 - High/Yes
How adequate is the data presentation?	4
Are units and terminology used correctly?	5 - High/Yes
Is the number of cases adequate?	4
Are the experimental methods/clinical studies adequate?	4
Is the length appropriate in relation to the content?	4
Does the reader get new insights from the article?	4
Please rate the practical significance.	4
Please rate the accuracy of methods.	4
Please rate the statistical evaluation and quality control.	3
Please rate the appropriateness of the figures and tables.	4
Please rate the appropriateness of the references.	5 - High/Yes
Please evaluate the writing style and use of language.	4

Please judge the overall scientific quality of the manuscript.	4
Are you willing to review the revision of this manuscript?	Yes

Comments to Authors:
Thanks for this interesting paper. There is definitely a lack of data concerning this very important „problem“ of many young doctors. Without doubt, this study will help to address everyday problems of young surgeons to hospital managers and politicians as it helps to objectify their needs. However, I think that some additional informations would improve the paper:

1.) It would be very interesting to have at least one control group within the study (i.e. 15 academic couples, who do not work as physicians) to unravel, if physicians do have special needs in comparison to „normal“ academic parents. This comparison might substantially help to address the problems of young surgeons.
2.) Within the discussion section (page 3) the authors hypothesize that „maternity and paternity leave in combination with the reported reduction of hours per week brings a relevant delay of surgical and medical treatment“. If possible, this hypothesis should be substantiated by concrete data. How long was the delay on average? Is it possible to extrapolate the financial costs of this delay? And (see 1.) is this a special problem of physicians (which would be a strong argument to demand more support)?
3.) It is necessary to know in how many different institutions the participants of this study were working. Please add this information to the methods section.
4.) Please add a column to table 1, which compares the „leave time“, the „reduction of hours worked per week“ and the „likert-scale“ in dependence on the gender.

Authors’ Response to Reviewer Comments

Mar 20, 2019

Reply to the comment of the editor

We appreciate the comment of the editor. Please find enclosed our reply to this comment.

Editor’s comment:
The main point is the lack of a “non-physician” control group. I understand the reviewers valid remarks, as I agree, that this would add to the article.
However, given the publication timeline of the special issue to which this article pertains; I understand that an additional survey might not be feasible.
This article is however of great value, so the lack of a control might just have to be listet as a limitation. After all this could serve as a first proof of concept, with the potential for a follow up article.

Authors’ reply:
We agree with the comment of the Editor. Accordingly, a limitations section was added to the revised version of the manuscript after the discussion (marked in yellow).

Reply to the comments of reviewer 1

We appreciate the comments of the reviewer. Please find enclosed our replies to these comments.

Reviewer’s comments 1-4:
The present study is well conducted and of good readability. There only remain some minor comments:
1. Title: The title is well chosen and quickly arouses interest, while also informative.
2. The abstract is within the desired word count and summarizes the manuscript in brief.
3. The introduction is concise and states the purpose of the study.
4. The layout of the study and the statistic tests applied are comprehensible and adequate. Selection of participants, study protocol and statistics are described.

Authors’ reply:
The authors appreciate the reviewer’s view that the present study is well conducted and of good readability. We thank the reviewer for the positive evaluation of title, abstract, introduction and layout.

Reviewer’s comment 5:
Interestingly the working hours were reduced in all couples except for one couple. Did this one couple consist of two surgeons or was this an ‘inter-professional’ couple? Also, did the mother or the father or both reduce the working hours?

Authors’ reply:
The couple without a reduction of working hours consisted of a surgeon and a non-surgeon. This information is now given in the results section of the revised manuscript (marked in yellow). We deliberately chose not to analyze whether the mother or the father were more likely to reduce their working hours as we did not want to provoke a gender equality discussion. Therefore, our questionnaire did not ask for the information, which partner reduced their working hours. However, we can say that in all cases it was only one of the partners who reduced. This information is also given in the results section of the revised manuscript (marked in yellow).

Reviewer’s comment 6:
In the discussion the authors state, that the use of professional child care exceeded the number of reported working hours. The conclusion, that the service hours of child care institutions do not match the working hours of physicians seems to be a reasonable point. Another factor could be the extra work load and working hours physicians especially in university hospitals face.

Authors’ reply:
We agree with the reviewer’s opinion that the additional work load in university hospitals might be another factor influencing the need for professional child care. However, as we did not ask the participants for details of their work load and working hours, we chose not to discuss this possible factor in the manuscript.
Reviewer’s comment 7:
References are clearly presented and represent the current state of literature. References should be formatted so that indent is equal.

Author’s reply:
We thank the reviewer for this comment. We reformatted the references in the revised version of the manuscript.

Reply to the comments of reviewer 2

We appreciate the fair and constructive criticisms of the reviewer. Please find enclosed our point-by-point reply to the reviewer’s comments.

First comment of the reviewer:
It would be very interesting to have at least one control group within the study (i.e. 15 academic couples, who do not work as physicians) to unravel, if physicians do have special needs in comparison to “normal” academic parents. This comparison might substantially help to address the problems of young surgeons.

Authors’ reply:
The reviewer is right that a control group is missing. However, our study has to been seen as a ‘proof of principle’, shedding light on the influence of parenthood on a medical career. We agree with the reviewer’s opinion that the comparison to a control group could be helpful for addressing the specific problems of young surgeons. The lack of a control group is listed as a limitation in the revised version of the manuscript (marked in yellow). The comparison to a control group is planned to be subject of a follow-up study.

Second comment of the reviewer:
Within the discussion section (page 3) the authors hypothesize that “maternity and paternity leave in combination with the reported reduction of hours per week brings a relevant delay of surgical and medical treatment”. If possible, this hypothesis should be substantiated by concrete data. How long was the delay on average? Is it possible to extrapolate the financial costs of this delay? And (see 1.) is this a special problem of physicians (which would be a strong argument to demand more support)?

Authors’ reply:
We appreciate the reviewer’s comment. We assume that not treatment (as stated in the comment), but training (as written in our manuscript) was meant. Unfortunately, we did not collect data on the participants’ state of training at the time of working hour reduction. As the mean reduction of hours worked per week was 30%, we can hypothesize that for any participant who has been in training when reducing the working hours, the mean delay was also 30%. This has been added to the discussion of the revised version of the manuscript (marked in yellow). An estimation of the financial costs is not possible because in Germany the salary depends on the level of training or specialization and the type of institution one is working at. This date has not been collected in our questionnaire.

Third comment of the reviewer:
It is necessary to know in how many different institutions the participants of this study were working. Please add this information to the methods section.

Authors’ reply:
The participants of the study were working in ten different institutions. This information is now given in the methods section of the revised manuscript (marked in yellow).

Fourth comment of the reviewer:
Please add a column to table 1, which compares the “leave time”, the “reduction of hours worked per week” and the “likert-scale” in dependence on the gender.

Authors’ reply:
Our study was deliberately designed to be gender-neutral. We aimed to analyze the impact of parenthood on the medical career of a couple and did explicitly not want to provoke a gender equality debate. Therefore, our questionnaire did not ask for information on whether the mother or the father reduced their working hours.

Editor Comments to Decision

Dear Authors,

thank you for submitting your revised manuscript to our special issue. As the article was already recommended to be accepted after minor revision by one reviewer and the other reviewer recommended revisions, that were clearly and adequately addressed as part of this revision I feel that this can now be accepted for publication.

The main point of concern of the reviewer recommending a major revision was the lack of a “non-physician” control group as part of the study. This is and remains a valid remark. It is now however thoroughly addressed and discussed by the authors as part of a limitations section highlighting the “proof of concept” character of this study. Even without the control group this article is of definite value to our special issue and should thus be published within the given, short time frame, as is.

Editor
